# High throughput sequencing from Angolan citrus accessions discloses the presence of emerging CTV strains

**DOI:** 10.1186/s12985-021-01535-x

**Published:** 2021-03-23

**Authors:** Aderito Tomàs Pais da Cunha, Michela Chiumenti, Laurindo Chambula Ladeira, Raied Abou Kubaa, Giuliana Loconsole, Vitantonio Pantaleo, Angelantonio Minafra

**Affiliations:** 1 Instituto Superior Politécnico do Kuanza Sul (ISPKS), Rua 12 de Novembro, Sumbe, Angola; 2 Institute for Sustainable Plant Protection - Consiglio Nazionale delle Ricerche (CNR), Via Giovanni Amendola 165/A, Bari, Italy; 3 Centro Nacional de Investigação Científica (CNIC), 201 Ho Chi Min Avenue, CP 34, Luanda, Angola

**Keywords:** Citrus tristeza virus, Angola, DsRNA, High-throughput sequencing, Coat protein, Virus strains

## Abstract

**Background:**

Citrus industry is worldwide dramatically affected by outbreaks of Citrus tristeza virus (CTV). Controls should be applied to nurseries, which could act as diversity hotspots for CTV. Early detection and characterization of dangerous or emerging strains of this virus greatly help to prevent outbreaks of disease. This is particularly relevant in those growing regions where no dedicated certification programs are currently in use.

**Methods:**

Double-stranded RNA extracted from Citrus spp. samples, collected in two locations in Angola, were pooled and submitted to a random-primed RNA-seq. This technique was performed to acquire a higher amount of data in the survey, before the amplification and sequencing of genes from single plants. To confirm the CTV infection in individual plants, as suggested by RNA-seq information from the pooled samples, the analysis was integrated with multiple molecular marker amplification (MMM) for the main known CTV strains (T30, T36, VT and T3).

**Results:**

From the analysis of HTS data, several assembled contigs were identified as CTV and classified according to their similarity to the established strains. By the MMM amplification, only five individual accessions out of the eleven pooled samples, resulted to be infected by CTV. Amplified coat protein genes from the five positive sources were cloned and sequenced and submitted to phylogenetic analysis, while a near-complete CTV genome was also reconstructed by the fusion of three overlapping contigs.

**Conclusion:**

Phylogenetic analysis of the ORF1b and CP genes, retrieved by de novo assembly and RT-PCR, respectively, revealed the presence of a wide array of CTV strains in the surveyed citrus-growing spots in Angola. Importantly, molecular variants among those identified from HTS showed high similarity with known severe strains as well as to recently described and emerging strains in other citrus-growing regions, such as S1 (California) or New Clade (Uruguay).

**Supplementary Information:**

The online version contains supplementary material available at 10.1186/s12985-021-01535-x.

## Background

Citrus tristeza virus (CTV) is still one of the major threats for citrus industry worldwide [1, 2]. Most of CTV introductions into new areas are due to budwood grafting, whereas aphid transmission is important for local spread in orchards. In Angola most of the citrus plantations are constituted of cultivated varieties (cvs.) Valencia late, Pera-Rio and Bahia (Washington navel) grafted on Poncirus trifoliata cv. “fly-dragon” and Citrus limon cv. Cravo rootstocks. Angola is one of largest African citrus producer country, with an estimated commercial production in 2019 of 415,146 tonnes on a dedicated surface of 38,413 ha (www.fao.org/faostat). Citriculture has been one of the fastest-growing industry in the last decade, which has an impact on the country's agricultural economy.

A previous report [3] described the presence of several CTV isolates in Angola and Sao Tomè e Principe, mostly affected by co-infection of diverse severe strains, sharing similarity with T3, T36 or B249, thus underlining further risks due to the introduction of propagative materials from abroad. The very wide array of phenotypical expression that CTV strains elicit on infected Citrus species and grafted combination, ranges from severe decline and dieback to mild or even latent response [2]. Therefore, in a citrus industry not ruled by the certification of propagative material, it could be common that budwood grafting actually spreads the infection. This inadvertent spread of CTV potentially severe strains can irreversibly compromise the nationwide production of citrus for fresh fruit, transformation and export trade.

In recent years, the application of high-throughput sequencing (HTS) to citrus virus-like infections—often associated with an unknown etiology—brought to the discovery or characterization of several new viruses [4–9]. The same approach (either through small RNA or dsRNA sequencing) has been used in the deep characterization of field citrus accessions affected by CTV in the aim of genotyping the viral population, surveying introduction and spread of potentially noxious strains, or controlling the evolution and persistence of cross protection isolates [10–15].

We tried the application of a sensitive and unbiased molecular diagnostic tool—like RNA-seq [16]—in a small survey of Angolan citrus industry, to test for the major presence of CTV and successfully detected several isolates which apparently span along all the increasing number of strains recently described in the CTV diversity.

## Methods

### Plant material, RNA extraction and RNA-seq

Eleven citrus sources (Table 1) were selected in late summer 2017 in a nursery (Luanda area) and in a private orchard (Quinta do Pomares, Kwanza Sul) due to the presence of virus-like symptoms, as chlorosis, interveinal yellowing, wrinkled leaf blade and slight dwarfing. In the orchard, plants showed slight alteration on the trunk, similar to stem-pitting and early fall of the fruits.

**Table 1:** Sample species collected and analyzed, collection place, molecular markers used in RT-PCR and GenBank accession numbers for cloned coat proteins

Sample species and isolate code	Location	T36CP^a^	T36pol^a^	VTpol^a^	T3K17^a^	Cloned CP codes and GenBank accession numbers
1A C. sinensis	Nursery	–				
1B C. sinensis	Nursery	–				
1C AO1 C. sinensis	Nursery	+	–	+	–	AO1.4, MW388800
2A C. limon Eureka	Nursery	–				
2B AO2 C. sinensis Bahia	Nursery	+	–	+	–	AO2.3, MW388801
2C AO3 C. reticulata	Nursery	+	–	–	+	AO3.1, MW388802
2D Lime Tahiti	Nursery	–				
3A AO4 C. sinensis Bahia	Orchard (Quinta do Pomares)	+	–	+	–	AO4.3, MW388803
4A AO5 C. sinensis Bahia	Nursery	+	–	+	–	AO5.1, MW388804; AO5.3, MW388805; AO5.4, MW388806
7A C. sinensis Bahia	Orchard (Quinta do Pomares)	–				
7B C. sinensis Bahia	Orchard (Quinta do Pomares)	–				

^a^Molecular markers as described in [22]

Lyophilized leaves (250 mg) from each of the eleven sources were extracted for the purification of double stranded RNA [17]. An average of about 10 ng/ml of purified nucleic acid was obtained through the cellulose-capture steps (included nuclease digestions) and final resuspension was in a volume of 15 µl. Upon normalization for an equal amount, aliquots of all the eleven extracted dsRNA were pooled up to reach a total of 500 ng. An anchored 12mer-random primer was used for the reverse transcription, employed with Superscript III kit (Life technologies, USA) [17]. The PCR amplification was done in 15 cycles. An Illumina sequencing of 150 nt-long reads in paired-ends was obtained for the library by service outsourcing at Genewiz (USA).

### Bioinformatics

After adapter sequence removal, the library was then submitted to quality checks, including a trimming based on quality (Q20) using fastx toolkit (http://hannonlab.cshl.edu/fastx_toolkit/). Finally, all reads were assembled by SPAdes software [18] and annotated by interrogation to BLASTn and BLASTx [19] through NCBI of nr nucleotides and RefSeq viral protein databases, respectively. E-score value thresholds, 10–6 for BLASTn and 10–4 for BLASTx, respectively, were applied to select significant hits. Contigs matching with CTV sequences (spanning from the largest one of 9,686 nt up to 2,000nt) were classified by size, genomic position and consistency of coding sequence (by ORFinder at NCBI; https: //www.ncbi.nlm.nih.gov/orffinder/) and, furthermore, their putative strain attribution was reported according to the first three hits of BLASTn. Additional smaller-than-2,000nt contigs were not considered for the analysis.

The alignments were done by MUSCLE algorithm embedded in MEGA X [20] and phylogenetic inference was derived using the Maximum Likelihood method with T92 + G substitution model and 1,000 bootstrapping replications. A similar approach, using a T92 + I substitution model, was followed to test the sequences of CTV ORF1b retrieved from 5 contigs (nodes 1, 4, 15, 20 and 39) that contained either a complete or partial coding sequence of this ORF, against the phylogenetic background of the same strain-specific isolates used for the CP gene. The alignments from both the sequence panels were analyzed for a pairwise identity matrix by SDT v. 1.2 [21].

### Reverse transcription-PCR and Sanger sequences

Since the annotation of HTS data predicted the presence of CTV contigs in the sequenced pool of the sampled citrus accessions, a multiple molecular marker analysis [22] was individually performed by RT-PCR amplification of all the eleven original dsRNA extracts used for HTS. The CP gene (full ORF), amplified only in five out of the eleven samples using the T36CP primer set, were cloned (using pSCa vector plasmid, Strataclone, USA) and at least three clones per sample were Sanger sequenced. Most of the other molecular marker sets [22] failed to amplify any fragment for the same samples, but VTPOL and T3K17 (see "Results" section).

The CP gene sequences, obtained from the cloned PCR products of the five CTV-infected samples, were aligned with the CP genes of the most representative strains retrieved through BLASTn and a phylogenetic analysis was performed. Most of the strain-specific reference sequences described in Yokomi et al. [15] were used in the analysis. All the CP genes reported by Silva et al. [3] from isolates of Angola and Sao Tomè e Principe were also included to have a comparison among isolates coming in a different time-frame from the same geographical region. Additionally, a selection of CTV CP gene sequences originated in South America was introduced in the analysis, following the hypothesis of the phylodynamic spread of a new strain [23].

## Results

### Identification of CTV contigs by RNA-seq

The sequencing output of the dsRNA library consisted of 39,704,476 reads with a mean quality score of 3355 and the 74.72% of bases called with QC ≥ 30. The de novo assembly of cleaned reads produced a total of 15,539 contigs with length ranging from 9366 to 102 nts. After the BLASTn search for homologies, the classification of viral-derived sequences resulted in a number of 176 contigs belonging to CTV (corresponding to 1.13% of total de novo assembled contigs) with an average depth of 42,083 X. The sequence of the recovered near-full genome spans 18,709 nucleotide length from the very beginning of ORF1 to the 3′end untranslated region (GenBank acc. nr. MW388809).

The 22 most relevant CTV contigs (> 2000 nt) were reported in Additional file 1 with their length, coverage, BLASTn results as nucleotide identity % over the first three hits and position on 1st matching hit genome. The majority of those contigs are located in the central genomic region (mostly inside the ORF1a/1b), in spite of the large representation of dsRNA of sub-genomic RNAs in the 3′ end portion, accumulated during the replication [24, 25], and only three of them were located in the 3′ end region (nodes 7, 25 and 46). Three large contigs (nodes 5, 4 and 7) turned out to be overlapping for 101 nt (node 5 vs. 4) and 99 nt (node 4 vs. 7), respectively; thus a reconstruction of a single, near-full length genome was possible.

A few contigs were annotated as Citrus endogenous pararetrovirus [26] and as Citrus yellow mosaic badnavirus, and these sequences most likely belong to introgressed pararetroviral remnants [27]. Only a single contig (375-nt long) matched to Citrus sudden death-associated virus (not shown). In the focus of the present work, these latter contigs were not taken in account for further elaborations. Unexpectedly, no viroid-like sequences were detected in the contigs or when reads were reference-mapped.

### Multiple Molecular Marker analysis

The amplification of MMM (to characterize isolates belonging to T30, T36, VT and T3 strains) and the sequences obtained from the CP gene clones shed a little light on the strain attribution of the five CTV-positive accessions (Table 1). While in all five sources the T36CP primers amplified the full length CP gene, four of them (AO1, AO2, AO4 and AO5) were also positive to VTPOL and only one (AO3) to T3K17 primers, respectively. No other set of primers listed in Hilf et al. [22] reacted positively. Indeed, a further in silico analysis of the CTV contig sequences revealed the absence of fully conserved length matches with any of those primers. Several of them were only partially embedded (in between of 11 to 16-nt recognizable stretches over the 22–24 oligonucleotide size) around their expected locations.

Overall, the sequences of seven CP gene clones were selected and deposited in GenBank (NCBI; acc. numbs. are reported in Table 1) as well as two more CP genes derived from the HTS contigs 7 and 25 (Additional file 1). One clone for each accession (AO1, AO2, AO3 and AO4) was reported since they were relatively homogeneous around a consensus sequence; for isolate AO5, three different clones were considered in further analysis due to their higher variability.

### Sequence analysis by SDT identity matrix

**Fig. 1:**
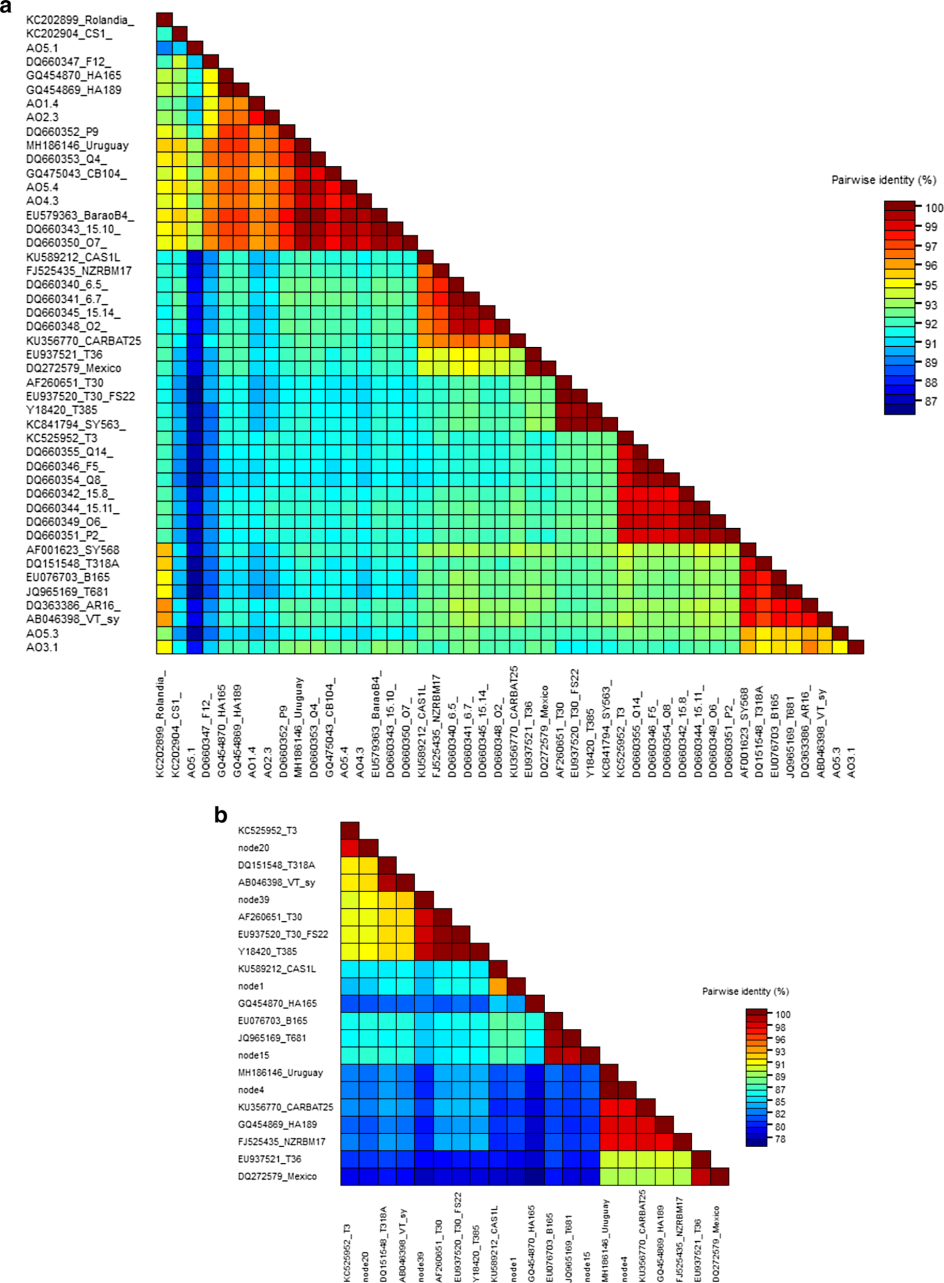
Identity matrix generated by aligning CTV sequences from Angolan isolates with those from reference strains retrieved by GenBank. Accession number and strain/isolate name is reported for each sequence. **a** Coat protein gene sequences; AO are the cloned coat proteins from Angolan CTV-positive sources. **b** ORF1b gene sequences; 'nodes' sequences represent the RNA-dependent RNA polymerase from the library contigs

The pairwise identity matrix, calculated for the CP gene sequences (Fig. 1a), showed the lowest value (86.6%) in the comparison between two CP clones from the same Angolan isolate (AO5.1 vs AO5.3). Although it is difficult to assess a unequivocal association between the only two CP sequences retrieved from the contigs 7 and 25 and those obtained by cloning the CP amplicons from CTV-positive sources, it is important to highlight the high nucleotide identity shared among two of them, 97.5% (node 25-CP vs AO5.3) and 99.0% (node 7-CP vs AO5.4).

The SDT identity matrix drawn for the ORF1b sequences (Fig. 1b), extrapolated from the HTS obtained contigs, gave a further contribution in demonstrating the strain differentiation. Some contigs from Angolan sources (like ORF1b sequences inside nodes 1, 4 and 15, which are, respectively, closer to S1, RB and VT strains) (see Additional file 1 for putative strain attribution), showed a low similarity value when compared with all main strains; node 1, as an example, ranged between 93.6% nt identity paired with S1 to as low as 78–79% when compared to isolates in the T36 group. The average identity among the RB-like contig (node 4) vs. T3- or T30-like contigs (nodes 20 and 39) reached a percentage as low as 80.3–82.1.

### Phylogenetic analysis

**Fig. 2:**
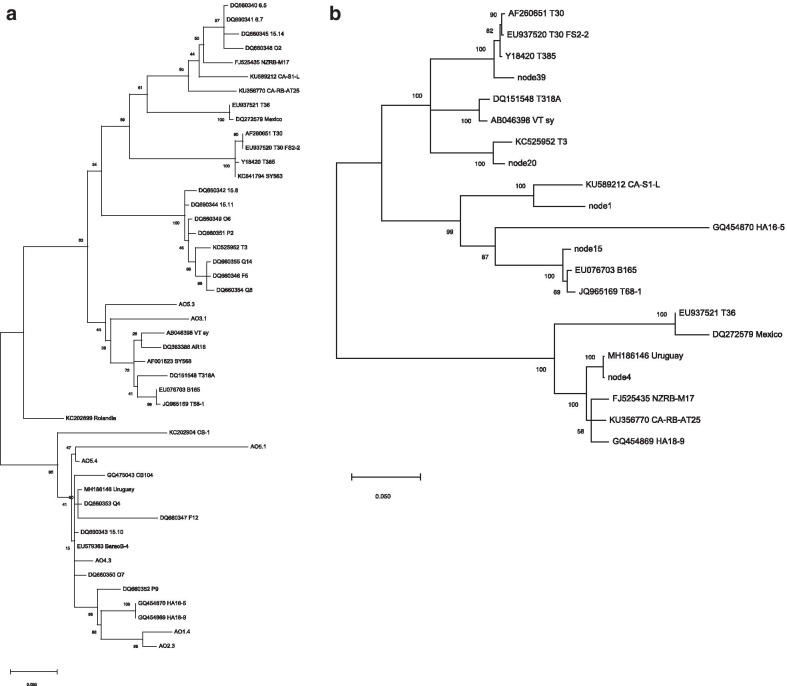
Maximum likelihood phylogenetic trees generated using the sequences from the CTV-positive samples from Angola and the homologous sequences retrieved from GenBank. Accession numbers, geographic origin or strain/isolate name is reported on the tips. **a** Coat protein gene; **b** ORF1b sequences; 'nodes' sequences represent the RdRp from the library contigs. Bootstrap value after 1000 replicates is indicated at each node. Bar unit of nucleotide distances is reported

The wealth of CP gene sequences in public datasets allowed a larger comparison in the phylogenetic analysis of Angolan isolates against a wide panel of well established and emerging strains. Figure 2a reports the topology of sequenced CP clones from Angolan CTV sources. Two clones (AO3.1 and AO5.3) cluster in the VT group, while the major set of sequences (AO4.3, AO5.4, AO2.3, AO1.4 and AO5.1) are grouped in a large cluster, which include the resistance-breaking isolate from Uruguay, and the Capao Bonito severe SP strain, Rolandia SP and BaraoB4 isolates from Brasil. This cluster could be potentially identified as the New Clade described by Benitez-Galeano et al. [23].

Some sequences reported by Silva et al. [3] in 2007 from isolates of Angola and Sao Tomè e Principe (DQ660350, DQ660343, DQ660347, DQ660353, DQ660352), also originated mainly from sweet orange sources, grouped in the same cluster to some of our clones (AO4.3, AO1.4 and AO2.3) and apart from the main established strains (T36, T30, T3, T68 and VT), suggesting that several isolates, most likely belonging to the monophyletic group identified by Benitez-Galeano et al. [23], are still present in Angolan citrus germplasm. Indeed, in the latter study [23], two CP cloned sequences from Angola were inferred in the phylodynamic design of the NC strain world-wide diffusion and supposed to enter Angola around the year 2002. These two Angolan isolates sequenced in 2007- DQ660350 and DQ660353—showed 99% identity to C257-9 (Argentina), B389-1 (South Africa) and DSST-13 (Uruguay), that are all RB-like isolates.

Figure 2b presents the phylogenetic tree that was inferred correlating the RdRp fragments contained in the contigs against the ORF1b of established reference strains. The five contig sequences considered (full ORF1b of about 1500 nt in nodes 4, 20 and 1 and partial coding sequences of about 1200 nt from nodes 39 and 15) spread in all the three main clusters. It is noteworthy the position of two contigs (node 39 and 20) in the T30 and VT group, while other two (node 1 and 15) map closely to the presumptive New Clade domain with T68, B165 and S1 strains.

Finally, as already noted, node 4 and node 7, included in the near-full genome, are gathered in the RB cluster, since node 4 sequence is almost identical to Uruguay DSST-17 and node 7 shows 99% nt identity with CTV isolates from South Africa, China and Brasil attributed to RB strain (Additional file 1). The other two large contigs (nodes 5 and 4), which overlap to compose the genome, both share not less than 99% identity, stably along all the genes, with isolates DSST-17 (MH186146, Uruguay), Crete 1825 (KF908013, Greece), B390-5 (KU883265, South Africa) and CA-RB-AT35 (KU358530, USA), that can be all listed in the RB-like group.

## Discussion

In the present work, we show that an unbiased, diagnostic application of HTS for the detection of noxious viruses affecting Citrus spp. even in a small survey, can sort, in a single analysis, an array of the extant diversity among CTV variants. Therefore, it has been possible to observe the high complexity of CTV isolates present in a few Angolan samples. This approach satisfactorily delivered a good quality and amount of genetic information, in a short time and reduced costs as pooled samples were processed, compared to lengthy PCR cloning and sequencing of sample by sample procedure.

Similarly, Zablocki and Pietersen [10] pointed the higher ability of HTS to detect minor variants than cloning efforts, due to the higher genome coverage and the viral population appeared to be more complex than previously suggested. Licciardello et al. [12] also reconstructed, by means of HTS, the phylogenetic relationships of two prevalent isolates found in Sicily with Mediterranean and exotic isolates. Importantly, bioinformatic screening on the CTV contig sequences obtained in our study revealed the absence of a whole conserved length that matched for most of the MMM primers applied according Hilf et al. [22]. Several of them were only partially embedded around their expected locations; but the consistent variability shown in their sequences certainly hampered the possibility to correctly anneal for a successful amplification. This evidence of mismatches for the molecular markers suggests a wide and recent appearance of recently discovered strains in the CTV population scenario. Most likely, genetic variability of these strains escapes the specificity of primers designed on multiple alignments, which are restricted to a batch of strains assumed as reference in the past [1]. Nevertheless, through the MMM amplification and cloning the five CTV-positive accessions were identified, at least as the more represented sequences in the clones, as VT- or T3-strain belonging isolates. This feature is supported by the evidence that 10 HTS contigs, out of 22 analysed, are also attributed to VT and T3 strains.

Conversely, the presence of isolates sharing high homology with new and emerging strains (like S1, RB and NC, largely and recently detected from South America and South Africa) reveals that their spread is enlarging and maybe in the past most of these isolates could have been hidden or misinterpreted in their strain attribution. We showed that several CTV sequences having a diversified strain-specific similarity are present—most likely in mixed infections—in our tested accessions. The accession AO5, among them, seems to be the main recipient of CTV molecular variants.

Silva et al. [3] already reported that, due to quick development of citrus industry in Angola, grafting on rootstocks imported from Brasil and South Africa was a common propagation technique. Therefore, the establishment of a national repository of virus-tested budwood germplasm would be highly desirable for a safe movement and spread of valuable citrus commercial cultivars.

## Conclusion

In recent years, propagation and trade of plants for plantings have expanded and diversified, increasing the risks of introducing pests to new geographical areas. In addition, this introduction and uncontrolled spread could elicit recombination events among different strains.

Here we have shown that in Angola there is a wide reservoir of diversity of CTV isolates, including severe strains. In the frame of an international strategy against the introduction and spread of aggressive viruses to prevent damages to crops and wild plants, the output of our work aims to stimulate more accurate plant virus surveys in Angola and in other African countries that are being integrated into the agro-industry international trades. Here we have applied a diagnostic HTS assay, along with a conventional PCR and cloning approach and phylogenetic analyses. For eradication management and potential application of cross protection control [28, 29], a wider picture of severe virus variants in larger regional districts is required. This effort would need either an intensive application of diagnostic tools and information technologies coupled to dedicated variant calling algorithms [30].

## Supplementary Information

**Additional file 1.** Major CTV contigs with position on the mapping genome and coverage depth. The first BlastN hits are reported with the % of nucleotide identity and a relative strain attribution is presented.

## Data Availability

The sequences analyzed during the current study are available in the NCBI GenBank repository under accession numbers: Coat protein sequences of CTV: from MW388800 to MW388808; near-full length CTV genome: MW388809.

## Abbreviations

CP: Coat protein
CTV: Citrus tristeza virus
dsRNA: Double-stranded RNA
HTS: High-throughput sequencing
MMM: Multiple molecular marker
ng: Nanograms
nt: Nucleotide
ORF: Open reading frame
RB: Resistance breaking
RefSeq: Reference sequence
RdRp: RNA-dependent RNA polymerase
RNA-seq: RNA sequencing
RT-PCR: Reverse transcription polymerase chain reaction

## References

[1] Harper SJ (2013). Citrus tristeza virus: evolution of complex and varied genotypic groups. Front Microbiol.

[2] Dawson WO, Bar-Joseph M, Garnsey SM (2015). Moreno PCitrus tristeza virus: making an ally from an enemy. Annu Rev Phytopathol.

[3] Silva G, Fonseca F, Santos C, Nolasco G (2007). Presence of citrus tristeza virus in Angola and São Tomé e Príncipe: characterization of isolates based on coat protein gene analysis. J Plant Pathol.

[4] Loconsole G, Önelge N, Potere O, Giampetruzzi A, Bozan O, Satar S, De Stradis A, Savino V, Yokomi RK, Saponari M (2012). Identification and characterization of Citrus yellow vein clearing virus, a putative new member of the genus Mandarivirus. Phytopathology.

[5] Loconsole G, Saldarelli P, Doddapaneni H, Savino V, Martelli GP, Saponari M (2012). Identification of a single-stranded DNA virus associated with citrus chlorotic dwarf disease, a new member in the family Geminiviridae. Virology.

[6] Roy A, Stone AL, Shao J, Otero-Colina G, Wei G, Choudhary N, Achor D, Levy L, Nakhla MK, Hartung JS, Schneider WL, Brlansky RH (2015). Identification and molecular characterization of nuclear Citrus leprosis virus, a member of the proposed Dichorhavirus genus infecting multiple Citrus species in Mexico. Phytopathology.

[7] Matsumura EE, Coletta-Filho HD, Nouri S, Falk BW, Nerva L, Oliveira TS, Dorta SO, Machado MA (2017). Deep sequencing analysis of RNAs from Citrus plants grown in a citrus sudden death-affected area reveals diverse known and putative novel viruses. Viruses.

[8] Navarro B, Minutolo M, De Stradis A, Palmisano F, Alioto D, Di Serio F (2018). The first phlebo-like virus infecting plants: a case study on the adaptation of negative-stranded RNA viruses to new hosts. Mol Plant Pathol.

[9] Navarro B, Zicca S, Minutolo M, Saponari M, Alioto D, Di Serio F (2018). A negative-stranded RNA virus infecting citrus trees: the second member of a new genus within the order Bunyavirales. Front Microbiol.

[10] Zablocki O, Pietersen G (2014). Characterization of a novel citrus tristeza virus genotype within three cross-protecting source GFMS12 sub-isolates in South Africa by means of Illumina sequencing. Arch Virol.

[11] Varveri C, Olmos A, Pina JA, Marroquìn C, Cambra M (2015). Biological and molecular characterization of a distinct *Citrus tristeza* virus isolate originating from a lemon tree in Greece. Plant Pathol.

[12] Licciardello G, Scuderi G, Ferraro R, Giampetruzzi A, Russo M, Lombardo A, Raspagliesi D, Bar-Joseph M, Catara A (2015). Deep sequencing and analysis of small RNAs in sweet orange grafted on sour orange infected with two Citrus tristeza virus isolates prevalent in Sicily. Arch Virol.

[13] Read DA, Pietersen G (2017). Diversity of Citrus tristeza virus populations in commercial grapefruit orchards in Southern Africa, determined using Illumina MiSeq technology. Eur J Plant Pathol.

[14] Yokomi RK, Selvaraj V, Maheshwari Y, Saponari M, Giampetruzzi A, Chiumenti M, Hajeri S (2017). Identification and characterization of citrus tristeza virus isolates breaking resistance in trifoliate orange in California. Phytopathology.

[15] Yokomi RK, Selvaraj V, Maheshwari Y, Chiumenti M, Saponari M, Giampetruzzi A, Weng Z, Xiong Z, Hajeri S (2018). Molecular and biological characterization of a novel mild strain of citrus tristeza virus in California. Adv Virol.

[16] Olmos A, Boonham N, Candresse T, Gentit P, Giovani B, Kutnjak D (2018). High-throughput sequencing technologies for plant pest diagnosis: challenges and opportunities. Bull. OEPP/EPPO Bull..

[17] Marais A, Faure C, Bergey B, Candresse T (2018). Viral double-stranded RNAs (dsRNAs) from plants: alternative nucleic acid substrates for high-throughput sequencing. Methods Mol Biol.

[18] Bankevich A, Nurk S, Antipov D, Gurevich A, Dvorkin M, Kulikov. SPAdes: a new genome assembly algorithm and its applications to single-cell sequencing. J Comput Biol 2012;19:455–477. 10.1089/cmb.2012.0021 PMC3342519 22506599

[19] Altschul SF, Madden TL, Schäffer AA, Zhang J, Zhang Z, Miller W, Lipman DJ (1997). Gapped BLAST and PSI-BLAST: a new generation of protein database search programs. Nucl Acids Res.

[20] Kumar S, Stecher G, Li M, Knyaz C, Tamura K (2018). MEGA X: molecular evolutionary genetics analysis across computing platforms. Mol Biol Evol.

[21] Muhire BM, Varsani A, Martin DP (2014). SDT: a virus classification tool based on pairwise sequence alignment and identity calculation. PLoS ONE.

[22] Hilf ME, Mavrodieva VA, Garnsey SM (2005). Genetic marker analysis of a global collection of isolates of Citrus tristeza virus: Characterization and distribution of CTV genotypes and association with symptoms. Phytopathology.

[23] Benítez-Galeano MJ, Castells M, Colina R (2017). The evolutionary history and spatiotemporal dynamics of the nc lineage of citrus tristeza virus. Viruses.

[24] Moreno P, Ambrós S, Albiach-Martí MR, Guerri J, Peña L (2008). Citrus tristeza virus: a pathogen that changed the course of the citrus industry. Mol Plant Pathol.

[25] Albiach-Martì MR, Romanowski V (2013). The complex genetics of citrus tristeza virus. Current issues in molecular virology: viral genetics and biotechnological applications.

[26] Roy A, Shao J, Schneider WL, Hartung JS, Brlansky RH (2014). Population of endogenous pararetrovirus genomes in Carrizo citrange. Genome Announc.

[27] Bhat AI, Hohn T, Selvarajan R (2016). Badnaviruses: the current global scenario. Viruses.

[28] Folimonova SY (2013). Developing an understanding of cross-protection by Citrus tristeza virus. Front Microbiol.

[29] Pechinger K, Chooi KM, MacDiarmid RM, Harper SJ, Ziebell H (2019). A New era for mild strain cross-protection. Viruses.

[30] Ghasemzadeh A, ter Haar MM, Shams-Bakhsh M, Pirovano W, Pantaleo V, Pantaleo V, Chiumenti M (2018). Shannon Entropy to evaluate Substitution Rate Variation Among Viral Nucleotide Positions in Datasets of Viral siRNAs. Viral Metagenomics Methods in Molecular Biology.

