# Supplementary material for: High throughput sequencing from Angolan citrus accessions discloses the presence of emerging CTV strains
Source: Virol J. 2021 Mar 23;18:62. doi: 10.1186/s12985-021-01535-x (PMC7988965; doi:10.1186/s12985-021-01535-x)
Supplement: Supplementary file 1 — Additional file 1. Major CTV contigs with position on the mapping genome and coverage depth. The first BlastN hits are reported with the % of nucleotide identity and a relative strain attribution is presented. [file 12985_2021_1535_MOESM1_ESM.docx]

**Additional Table 1 -** Major CTV contigs with position on the mapping genome and coverage depth. The first BlastN hits are reported with the % of nucleotide identity and a relative strain attribution is presented.

| **Node** | **Length** | **Coverage (X)** | **Position on 1^st^ Blast hit genome** | **Hits** | **Hit sequence origin** | **Nucleotide identity (%)** | **Putative strain attribution** |
| --- | --- | --- | --- | --- | --- | --- | --- |
| 1 | 9366 | 335.862 | 1909-11234 | KU589213 CA-S1-L65  MH593380 CT91-A1 | USA  China | 91.53  91.21 | S1 |
| 2 | 9248 | 385.101 | 2138-11381 | KU883267 LMS6-6  GQ454870 HA16-5  JX266713Pum/M/T5 | South Africa  Hawaii  Taiwan | 99.75  99.20  96.20 | New Clade-UY |
| 4 | 6849 | 231.902 | 6973-13817 | MH051717 ,B389-1  MH186146 DSST-17  KU883265 B390-5 | South Africa  Uruguay  South Africa | 99.87  99.85  99.84 | RB |
| 5 | 6565 | 892.457 | 510-7074 | MH186146 DSST-17  KU883265 B390-5  KF908013 Crete1825 | Uruguay  South Africa  Greece | 99.76  99.76  99.65 | RB |
| 7 | 5499 | 165.153 | 13719-19217 | MH051717 B389-1  MH558665 CN-RB-9  KY110737 CSL01 | South Africa  China  Brasil | 99.55  99.49  99.47 | RB;**(acc. nr. CP gene** **MW388807)** |
| 11 | 4225 | 133.40 | 2624-7847 | KJ790175 Mac39  KC748392 SG29  KY110738 CSL02 | Italy  Italy  Brasil | 98.56  98.53  98.30 | VT |
| 15 | 3967 | 209.957 | 9550-13516 | MK033511 GFMS12-8  MK033510 GFMS12-1.3  KC333868 CT-ZA3 | South Africa  South Africa  South Africa | 98.19  98.19  98.19 | VT |
| 18 | 3746 | 292.942 | 4104-7844 | KC262793 L192GR  KU883266 Maxi  FJ868797 B165 | Greece  South Africa  India | 98.34  98.32  98.18 | VT |
| 19 | 3655 | 137.253 | 4593-8238 | KC333868 CT-ZA3  MK033510 GFMS12-1.3  MK033511 GFMS12-8 | South Africa  South Africa  South Africa | 99.09  99.07  99.01 | T68 |
| 20 | 3615 | 328.404 | 7543-11157 | MH051719 T3-KB  EU857538 SP  KC525952 T3 | South Africa  NewZealand  USA | 98.67  98.42  97.93 | T3 |
| 23 | 3489 | 746.596 | 54-3543 | KU589213CA-S1-L65  KU589212CA-S1-L  MK033511GFMS12-8 | USA  USA  South Africa | 91.57  91.57  90.64 | S1 |
| 24 | 3377 | 433.058 | 104-3469 | MK033511GFMS12-8  KC333868 CT-ZA3  MK033510 GFMS12-1.3 | South Africa  South Africa  South Africa | 95.13  95.07  95.01 | T68 |
| 25 | 3369 | 42.2959 | 13707-17070 | KY110738 CSL02  KC748392 SG29  KJ790175 Mac39 | Brasil  Italy  Italy | 99.02  98.90  98.84 | VT; **(acc. nr. CP gene** **MW388808)** |
| 29 | 3092 | 1427.46 | 3604-6695 | KY110738 CSL02  HM573451 Kpg 3  DQ151548 T318A | Brasil  India  Spain | 91.85  91.69  91.49 | VT |
| 32 | 3050 | 111.367 | 180-3227 | KC748391 Bau282  MH279618 702 5a  Y18420 T385 | Italy  USA  Spain | 97.74  97.67  97.60 | T385 |
| 35 | 2880 | 342.321 | 13449-16328 | KU883267 LMS6-6  KJ914662 Kat1  HQ912022 D1 | South Africa  India  India | 99.79  99.06  98.96 | T3 |
| 39 | 2658 | 30.5092 | 7923-10566 | KC517491 FS703-T30  KC748391 Bau282  KC517490 FL278-T30 | USA  Italy  Italy | 98.45  98.41  98.37 | T30 |
| 41 | 2602 | 31.9648 | 2661-5257 | AY995566 464-2  AY995565 464-1  AY995564 425 | USA  USA  USA | 97.35  97.31  97.31 | -- |
| 46 | 2445 | 98.7628 | 16855-19252 | KU883266 Maxi  MH051719 T3-KB  KC525952 T3 | South Africa  South Africa  USA | 98.25  98.08  97.83 | T3 - VT |
| 58 | 2245 | 210.43 | 7589-9821 | KC517493 FL202-VT  KC517494 FS701-VT  KC517492. FS703-VT | USA  USA  USA | 98.84  98.75  98.75 | VT |
| 60 | 2223 | 414.142 | 1501-3724 | KJ790175 Mac39  KC748392 SG29  AF001623 SY568 | Italy  Italy  USA | 98.88  98.88  98.16 | VT |
| 71 | 2096 | 42.5118 | 11703- 13798 | KC517489 FS701-T30  AF001623 SY568  MK779711. N4 | USA  USA  China | 95.94  95.85  95.71 | T30 |
